# 

**Published:** 1879-01

**Authors:** 


### CONTENTS.

ORIGINAL.

ART. I.—Umbilical Hernia in the Adult. By William Warren Potter, M. D., of Batavia, 200
ART. II.—Medical Society of the County of Albany. Semi-Annual Meeting, Nov. 6, 1878. Reported by T. K. Perry, M. D., Secretary, ........ .............................. 219

MISCELLANEOUS.

United States Census Letter from Joseph K. Barnes, Surgeon General U. S. Army, ... 226
Treatment of Diphtheria,.................. .. 228
A New Nerve,........ ..... ........ ...... 229
Proposed National Health Board,............ 230
An Eulogium on Doctors,.................... 232
English Quarantine,.......... .............. 232

EDITORIAL.

"Happy New Year,"............. .......... 233
Index Medicus,............................. 234
Death of Dr. H. T. Babcock,....... ........ 235
Popular Science Monthly,............ .. .... 235
Uranine,........ . ................ ........ 235

BOOKS REVIEWED:

Elementary Quantitive Analyses,......... ... 236
Ziemssen's Cyclopedia, vol. xvii,.......... 236
On the Therapeutic Forces,............... 237
Transactions of Medical Society, West Virginia,.. ......................... ........ 238

Books and Pamphlets Received,.............. 240

